# P-1664. Shifting Susceptibilities and Clinical Outcomes of Oncology Patients with *Streptococcus mitis*/*oralis* Bacteremia

**DOI:** 10.1093/ofid/ofae631.1830

**Published:** 2025-01-29

**Authors:** Guy Handley, Shivan Shah, Rebecca Bruning, Ju Hee Katzman, John Greene, Yanina Pasikhova

**Affiliations:** University of South Florida Morsani College of Medicine, Tampa, Florida; University of South Florida, Palmetto, Florida; Moffitt Cancer Center, Tampa, Florida; University of South Florida / Moffitt Cancer Center, Tampa, Florida; Moffitt Cancer Center, Tampa, Florida; Moffitt Cancer Center, Tampa, Florida

## Abstract

**Background:**

*Streptococcus mitis*/*oralis* cause most viridans group infections in oncology patients and may cause more severe clinical disease. Antimicrobial resistance to beta-lactams is not uncommon, and patients, including those with severe neutropenia, may be at higher risk. We sought to describe changes in susceptibility patterns of *S. mitis/oralis* and clinical characteristics in these patients.

**Figure:**
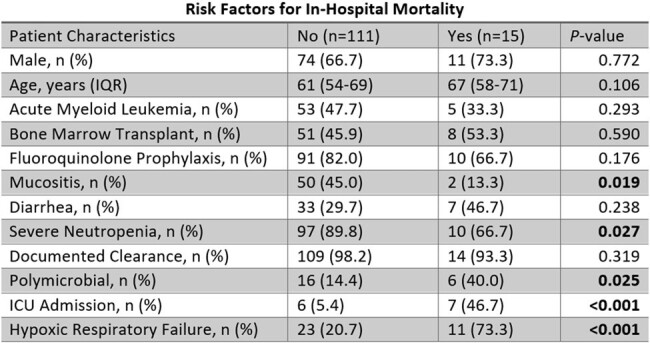

**Methods:**

Patients at an NCI-designated comprehensive cancer center from 01/01/2022-03/15/2024 with *S. mitis/oralis* in blood cultures were identified. Clinical and laboratory data were evaluated to describe susceptibility patterns and clinical outcomes.

**Figure:**
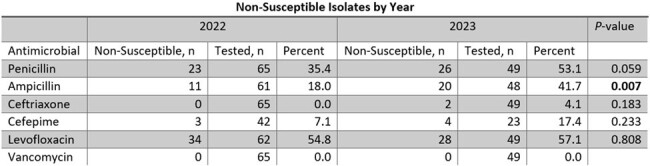

**Results:**

There were 126 patients included in the study. 58 (46%) had AML and 59 (46.8%) had undergone bone marrow transplantation. Severe neutropenia at the onset of bacteremia was present in 107 (84.9%). The median time to clearance was 1 day (IQR 1-2). Antibacterial prophylaxis was prescribed in 105 (83.3%), most of which was fluoroquinolones (96.2%). Polymicrobial bacteremia occurred in 22 (17.5%) patients. Hypoxic respiratory failure developed in 34 (27%) and 13 (10.3%) required ICU admission. In-hospital mortality occurred in 15 (11.9%) patients. Non-susceptibility was common for penicillin (41.6%) and levofloxacin (54.9%) but rare for ceftriaxone (2.4%) and vancomycin (0%). Ceftriaxone non-susceptibility was associated with cefepime non-susceptibility in all cases (3/3, 100%), while 6 (8.2%) isolates susceptible to ceftriaxone were non-susceptible to cefepime (p=0.001). Of 98 patients receiving fluoroquinolone prophylaxis, levofloxacin prophylaxis had a greater association with levofloxacin non-susceptibility than ciprofloxacin prophylaxis (86.7% vs 54.4%; p=0.002). Patients receiving ciprofloxacin prophylaxis had similar rates of levofloxacin non-susceptibility to those not receiving ciprofloxacin prophylaxis (54.4% vs 55.6%; p=0.900). Beta-lactam non-susceptible rates rose over time and were statistically higher for ampicillin in 2023 compared to 2022 (41.7% vs 18.0%; p=0.007).

**Figure:**
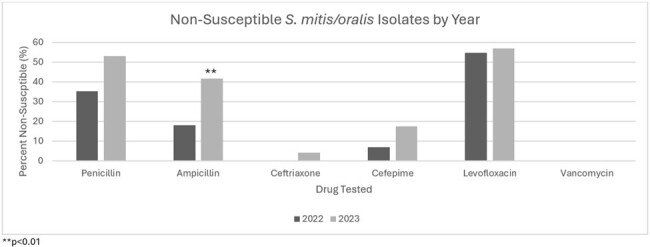

**Conclusion:**

Beta-lactam non-susceptibility among *S. mitis/oralis* isolates may be increasing. Cefepime susceptibility cannot be inferred from ceftriaxone susceptibility.

**Disclosures:**

**All Authors**: No reported disclosures

